# Comparison of an exercise program with and without manual therapy for patients with chronic neck pain and upper cervical rotation restriction. Randomized controlled trial

**DOI:** 10.7717/peerj.12546

**Published:** 2021-11-24

**Authors:** Jacobo Rodríguez-Sanz, Miguel Malo-Urriés, María Orosia Lucha-López, Carlos López-de-Celis, Albert Pérez-Bellmunt, Jaime Corral-de-Toro, César Hidalgo-García

**Affiliations:** 1 Faculty of Medicine and Health Sciences. ACTIUM Anatomy Group. Universitat Internacional de Catalunya, Sant Cugat del Vallès, Barcelona, Spain; 2 Department of Physiatry and Nursing. Physiotherapy Research Unit. Faculty of Health Sciences, Universidad de Zaragoza, Zaragoza, Spain; 3 Fundació Institut Universitari per a la recerca a l’Atenció Primària de Salut Jordi Gol i Gurina, Barcelona, Spain

**Keywords:** Physical therapy, Manipulation, Physiotherapy, Training, Neck pain

## Abstract

**Background:**

Cervical exercise has been shown to be an effective treatment for neck pain, but there is still a need for more clinical trials evaluating the effectiveness of adding manual therapy to the exercise approach. There is a lack of evidence on the effect of these techniques in patients with neck pain and upper cervical rotation restriction.

**Purpose:**

To compare the effectiveness of adding manual therapy to a cervical exercise protocol for the treatment of patients with chronic neck pain and upper cervical rotation restriction.

**Methods:**

Single-blind randomized clinical trial. Fifty-eight subjects: 29 for the Manual Therapy+Exercise (MT+Exercise) Group and 29 for the Exercise group. Neck disability index, pain intensity (0–10), pressure pain threshold (kPa), flexion-rotation test **(°),** and cervical range of motion **(°)** were measured at the beginning and at the end of the intervention, and at 3-and 6-month follow-ups. The MT+Exercise Group received one 20-min session of manual therapy and exercise once a week for 4 weeks and home exercise. The Exercise Group received one 20-min session of exercise once a week for 4 weeks and home exercise.

**Results:**

The MT+Exercise Group showed significant better values post-intervention in all variables: neck disability index: 0% patient with moderate, severe, or complete disability compared to 31% in the Exercise Group (*p* = 0.000) at 6-months; flexion-rotation test (*p* = 0.000) and pain intensity (*p* = 0.000) from the first follow-up to the end of the study; cervical flexion (*p* = 0.002), extension (*p* = 0.002), right lateral-flexion (*p* = 0.000), left lateral-flexion (*p* = 0.001), right rotation (*p* = 0.000) and left rotation (*p* = 0.005) at 6-months of the study, except for flexion, with significative changes from 3-months of follow up; pressure pain threshold from the first follow-up to the end of the study (*p* values range: 0.003–0.000).

**Conclusion:**

Four 20-min sessions of manual therapy and exercise, along with a home-exercise program, was found to be more effective than an exercise protocol and a home-exercise program in improving the neck disability index, flexion-rotation test, pain intensity, and pressure pain threshold, in the short, medium, and medium-long term in patients with chronic neck pain and upper rotation restriction. Cervical range of motion improved with the addition of manual therapy in the medium and medium-long term. The high dropout rate may have compromised the external validity of the study.

## Introduction

Chronic neck pain prevalence ranges from 5.9% to 38.7% and it is more common in women than in men (Popescu & Lee, 2020). This pathology is described as pain located between the occiput and the third thoracic vertebra that persists for more than 3 months (Côté, Cassidy & Carroll, 1998).

Cervical exercise has been shown to be an effective treatment for neck pain (Kay et al., 2005, 2009; Celenay, Akbayrak & Kaya, 2016). In a systematic review, Blomgren et al. (2018) concluded that training that involves deep muscles and global movement exercises is necessary to improve function and reduce symptoms in patients with neck pain (Blomgren et al., 2018). These programs combine submaximal effort exercises for the deep cervical muscles to improve coordination and motor control, and after exercises for the superficial cervical muscles to improve the ability of the neck to move in the whole range of movement. Deep muscle exercises consist of a low load movement of the head to the inner range of cranio-cervical flexion (Borisut et al., 2013).

A number of articles have examined whether a manual therapy approach should be added to cervical exercise protocols for neck pain (Miller et al., 2010; Celenay, Akbayrak & Kaya, 2016; Hidalgo et al., 2017). While a few studies have contended that adding manual therapy to exercise does not produce any added beneficial effect (Hidalgo et al., 2017), there is still a need for more clinical trials evaluating the efficacy of using a manual therapy and exercise approach on participants with neck pain with an indication of manual therapy, and more specifically with upper cervical rotation restriction. Upper cervical dysfunction could limit the efficacy of cervical exercise in these patients, as the main movement for training the deep neck flexor muscles comes from the upper cervical spine (Falla et al., 2003). Moreover, more than 60% of cervical axial rotation occurs in the upper cervical spine (Hidalgo García et al., 2016), a fundamental region for cervical function.

Lack of mobility and symptoms arising from upper cervical spine are considered to be the main indication for upper cervical manual therapy approach (Kaltenborn, 2012; Hidalgo García et al., 2016; Malo-Urriés et al., 2017; González-Rueda et al., 2021; Rodríguez-Sanz et al., 2021).

The effect of cervical exercise with and without manual therapy in patients with chronic neck pain and upper cervical rotation restriction is currently unknown (Aker et al., 1996; Beltran-Alacreu et al., 2015; Celenay, Akbayrak & Kaya, 2016).

Based on this, our hypothesis is that adding a manual therapy approach to a cervical exercise protocol for the treatment of patients with chronic neck pain and upper cervical rotation restriction is better than not adding manual therapy to improve pain, disability, and cervical range of motion.

The objective of this study is to compare the short (end of the intervention), medium (3 months), and medium-long (6 months) term effectiveness of adding a manual therapy approach to a cervical exercise protocol for the treatment of patients with chronic neck pain and upper cervical rotation restriction in pain, disability, and cervical range of motion.

## Materials & methods

The CONSORT statement has been followed.

### Study design

A single-blind randomized clinical trial was conducted. A researcher not involved in the study performed the randomization using Microsoft Excel 2010. Assignments were placed in a concealed opaque envelope and participants were randomly assigned to intervention groups.

The design was carried out in facilities at the University of Zaragoza, Spain (Clinicaltrials.gov number: NCT03670719). This study complied with the ethical principles for research on human beings as per the Declaration of Helsinki (Fortaleza, Brazil, October 2013). It was approved by the local ethics committee: “Comité Ético de Investigación Clínica de Aragón” (CEICA, no. 13/2018) and written consent was obtained.

### Subjects

Fifty-eight volunteer subjects were recruited (17 men, 41 women). This was the total number of patients referred by doctors over a 4-week period. The inclusion criteria were as follows: a medical diagnosis of chronic neck pain with more than 3 months of evolution (Miller et al., 2010); a positive result in the flexion-rotation test (less than 32° or a difference of 10° or more between the two rotations) (Hall & Robinson, 2004; Ogince et al., 2007; Blanpied et al., 2017); hypomobility in one or more segments of C0-1, C1-2, or C2-3 through manual assessment according to Zito, Jull & Story (2006) and Kaltenborn (2012) being over 18 years old; and having signed the informed consent. Exclusion criteria were: contraindications for manual therapy or exercise; having participated in exercise or manual therapy programs in the last 3 months; presenting warning signs or having suffered a relevant neck trauma (Rushton et al., 2014); an inability to maintain supine position; the use of pacemakers, as the magnets in the Cervical Range of Motion (CROM) device could alter their signal; an inability to perform a flexion-rotation test; language difficulties for the understanding of informed consent, exercises, or measurements; and pending litigation or lawsuits (González Rueda et al., 2017).

### Measurements

The primary outcome measures in this study were the neck disability index and the flexion-rotation test. Secondary outcome measures were pressure pain threshold, cervical range of motion, and pain intensity.

The neck disability index is a self-administered questionnaire with 10 sections, each with six possible answers representing six progressive levels of functional disability, 0 being the lowest level and five being the highest level in each section. Total scores ranged from 0 to 50 points, with higher scores indicating greater disability. The translation to Spanish has been validated (Andrade Ortega, Martínez & Ruiz, 2008). The internal consistency of the questionnaire was high (Cronbach alpha in the first administration: 0.89). The convergent validity in different clinical groups was excellent and it was sensitive to change. The questionnaire showed an excellent test-retest reliability (ICC 0.97) (Andrade Ortega, Martínez & Ruiz, 2008; González Rueda et al., 2017). In this study, the results of the questionnaire have been classified according to the following categories to have a direct clinical implication of the outcomes: 0–4 = no disability; 5–14 = mild; 15–24 = moderate; 25–34 = severe; and above 34 = complete (Vernon & Mior, 1991).

The flexion-rotation test was used to measure the upper cervical spine rotation. When performing the test, the subject was in supine and the evaluator passively took the patient’s cervical spine to its maximum flexion, and then rotated the head to the right and left sides with the occiput resting against the evaluator’s abdomen. The movement stopped when either the subject presented symptoms, or the evaluator found a firm end-feel, whichever happened first (Hall & Robinson, 2004; Malo-Urriés et al., 2017). A CROM device (floating compass; Plastimo Airguide, Inc, Buffalo Groove, IL, USA) was used, and three measurements were taken for each rotation and the mean value was used (González Rueda et al., 2017). The intratester reliability of this test has been established as ICC = 0.95 for right rotation and ICC = 0.97 for left rotation (Hall et al., 2010).

The pressure pain threshold was measured using a digital algometer (Somedic AB Farsta, Somedic SenseLab AB, Sösdala, Sweden) with a round surface area of 1 cm^2^, pressure was applied at a speed of 1 kg/cm^2^/s perpendicular to the skin. With the subject in supine, pressure was assessed over three points bilaterally: Trapezius, levator scapulae, and C5-6 zygapophyseal joint. The intensity of pain in the levator scapulae muscle was measured anterior to the point of the trapezius, at the location of the levator scapulae belly (Lluch et al., 2013). The intensity of pain in C5-6 zygapophyseal joint was measured lateral to the spinous process of C5, through the esplenio cervicis muscle belly (Lluch et al., 2013). Patients were instructed to press the button on the digital algometer the moment the sensation of pressure changed into pain. The mean results for the three trials and for each of the pressure points were calculated and used for analysis. The pressure pain threshold measurements have a high intratester reliability (ICC > 0.974) (Zamani et al., 2017).

Active cervical range of motion was measured in all cardinal planes for the assessment of general cervical mobility. For the active mobility testing, patients were asked to sit upright. Patients were asked to move their head as far as they could without pain (Lantz, Chen & Buch, 1999). A CROM device (floating compass; Plastimo Airguide, Inc, Buffalo Groove, IL, USA) was used. Intratester ICC values were established as being from 0.98 to 0.99 (Williams et al., 2012). Three measurements were taken for each movement and the mean value was calculated (González Rueda et al., 2017).

For the pain intensity evaluation, each subject reported his/her intensity of neck pain using the verbal Numeric Pain Rating Scale (NPRS) (scale: 0 = no pain/10 = worst pain) (Jensen, Karoly & Braver, 1986). The NPRS has shown consistency across different patient populations. The scale has a high reliability of 0.76 in patients with chronic neck pain. The minimum detectable change (MDC) was determined as two points, and minimal clinically important difference as 1.3 points (Cleland, Childs & Whitman, 2008).

The same researcher specialized in physical therapy, with training in evaluation and more than 5 years’ clinical experience, took the measurements before (T0), at the end of the intervention (T1), after 3 months (T2), and after 6 months (T3). This researcher remained blinded to each patient’s assignment group throughout the process. Study recruitment was then complete, and another researcher proceeded with the treatment approach according to the group assigned by the randomization process.

### Intervention

The intervention was administered individually in the facilities of the Universidad de Zaragoza. Participants in both groups received one 20-min session once a week for 4 weeks. The treatment was applied by a researcher with more than 5 years’ experience in physical therapy. Moreover, all patients were advised to perform the same exercises they had done in the clinic between two and five times a day every day, starting after the first session (Jull et al., 2002; Hansen et al., 2011; Falla et al., 2013; Gallego-Izquierdo et al., 2016). A weekly video call was made to monitor their adherence to these recommendations.

#### Exercise group

The exercise program was developed following the guidelines provided by Fernández-de-las-Peñas (Fernandez Peñas & Cleland, 2011). Each exercise session was composed of two sets of 10 repetitions, holding each exercise for 10 s, with a 40-s rest period between reps, and a 2-min rest between sets (Fernandez Peñas & Cleland, 2011).

**Figure 1:**
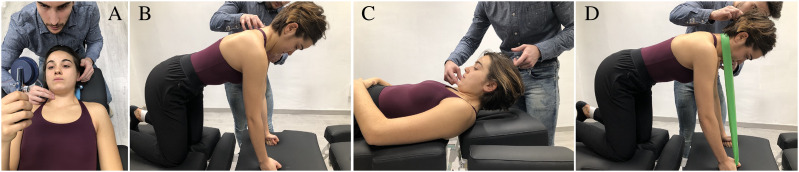
(A) Deep neck flexor. Subjects were instructed to “gently nod their head as though they were saying yes”. The physical therapist identified the target level that the subject could hold steadily for 10 s without use of the superficial neck flexor muscles using the craniocervical flexion test. Contribution from the superficial muscles was monitored using observation and palpation. Training was started at the target level the subject was able to achieve with a correct craniocervical flexion. The subjects were taught to perform a slow and controlled craniocervical flexion action. For each target level, the contraction lasted for 10 s, and the subject trained until able to perform 10 repetitions. At this stage, the exercise moved to the next target level^16^. (B) Deep neck extensors. The patient tucked their chin towards their chest slightly, and maintaining their chin in this position while gently extending their neck^20^. (C) Craniocervical flexion. Subjects were instructed to initiate the movement with a deep neck flexor contraction. Then they flexed the whole cervical column by lifting the head off the table^7^. (D) Extensors with resistance. The exercise is performed in a way similar to exercise “B” adding an external resistance to increase the intensity of the exercise^20^.

Patients started with the first treatment session performing cervical stabilization exercises in supine position. They were taught to perform the contraction of deep neck flexor muscles with a Stabilizer Pressure Biofeedback Unit (Chattanooga, TN, USA) (Jull, O’Leary & Falla, 2008; Celenay, Kaya & Akbayrak, 2016) (Fig. 1A) (Fernandez Peñas & Cleland, 2011). In the second session, the deep neck extensors were performed in a quadruped position (Fig. 1B) (Fernandez Peñas & Cleland, 2011). Patients had to perform a segmental extension movement from a position with the head bent down onto their chest in a horizontal direction (Fernandez Peñas & Cleland, 2011). In the third and fourth sessions, the patient trained craniocervical flexion in supine (Fig. 1C) by lifting the head off the table while keeping the spine in a neutral position (Fernandez Peñas & Cleland, 2011). The patient also activated the extensors by applying external resistance in a quadruped position (Fernandez Peñas & Cleland, 2011) (Fig. 1D). If a patient was not able to do an exercise, it was slightly adapted so that they could do it.

#### MT+exercise group

**Figure 2:**
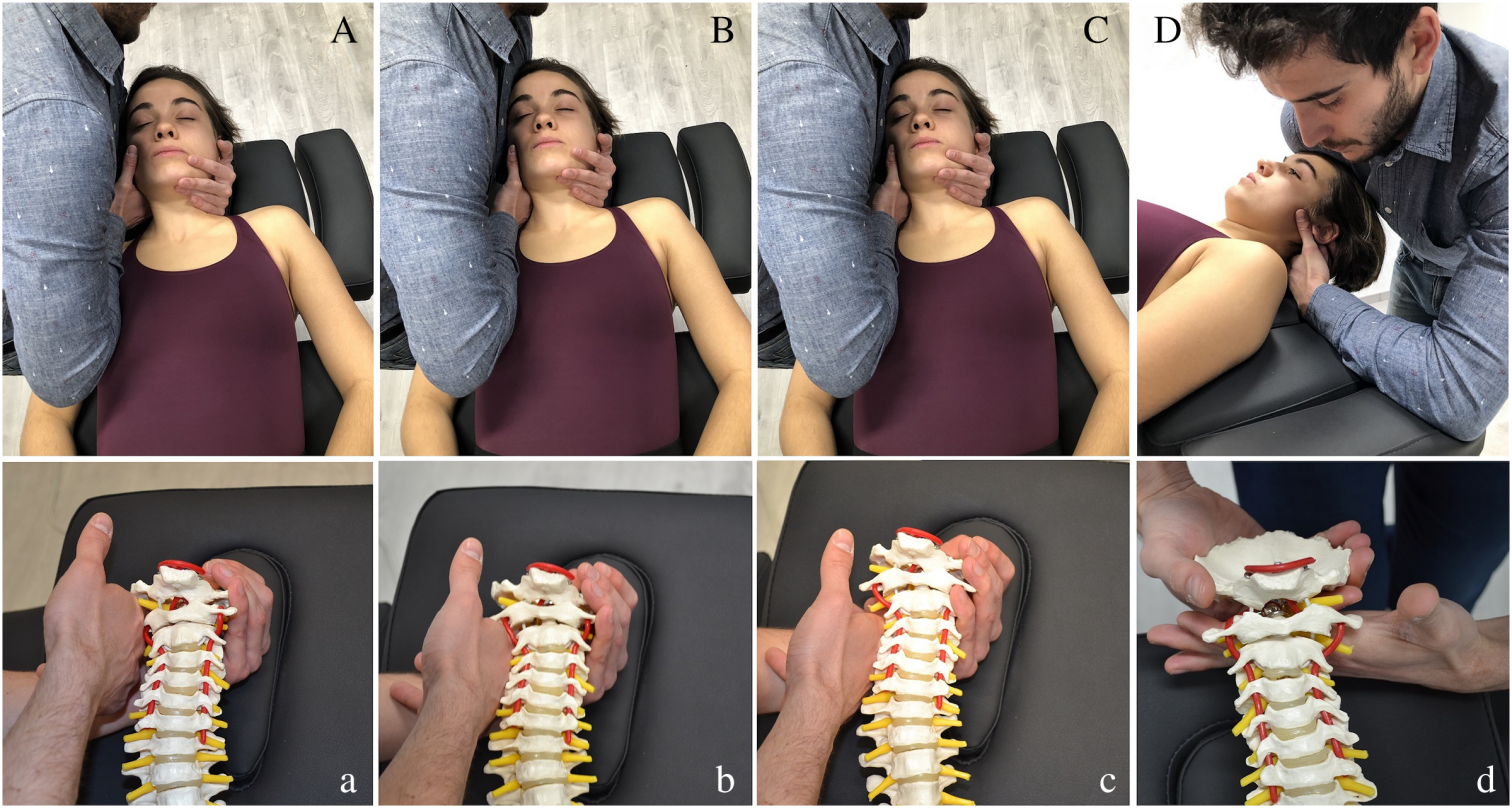
(Aa) C0-1 Traction manipulation in the resting position. Patient was in supine with neck in neutral position. The therapist gently cupped the patient’s chin with their hand while their arm was cradled around the head. The other hand placed the radial side of the index finger under the mastoid process and aligned the forearm in the line of drive pointing cranially. Then the therapist applied a cranial thrust^41,20,8^. (Bb) C1-2 Traction manipulation in the resting position. The same handling procedure was performed but the grip was relocated in the atlas vertebrae^41,20,8^. (Cc) C2-3 Traction manipulation in the resting position. The same handling procedure was performed but the grip was relocated in C3 vertebrae^41,20,8^. (Dd) C0-1 Dorsal mobilization. Patient was positioned in supine, with neck in neutral position. The therapist placed a hand dorsally at the level of the vertebral arch of C1 with the metacarpophalangeal and radial border of the index finger. The other hand was placed posteriorly under the occiput, with the shoulder positioned anteriorly on the patient’s forehead. The mobilization force was directed dorsally from the shoulder until the therapist felt a marked resistance, and then slightly more pressure was applied to perform a stretching mobilization^46,34,40^.

Manipulation (high velocity, low amplitude) of occipital-atlas (C0-1), atlas–axis (C1-2) and axis-C3 (C2-3) segments (Figs. 2Aa–2Cc), and/or dorsal mobilization (low velocity, high amplitude) of C0-1 segment (Fig. 2Dd), were combined with cervical exercise in all sessions (Kaltenborn, 2009, 2012; Krauss & Evjenth, 2009; Hidalgo García et al., 2016; Malo-Urriés et al., 2017). Manipulations were applied in the direction of traction, with the head in a neutral position (Carrasco-Uribarren et al., 2020). A maximum of two trials at each level were performed on each side, yielding 2–6 thrusts per each visit (Reynolds et al., 2020). Mobilization was performed for 5 min using repeated cycles of 45 s of mobilization and 15 s of rest (Hidalgo García et al., 2016; Malo-Urriés et al., 2017). The manual therapy techniques depended on patient’s segmental hypomobility, and the aim was to restore the mobility of the upper cervical joints. The same intervention was performed on every patient in the four manual therapy sessions. All the techniques followed the International Federation of Orthopaedic Manipulative Physical Therapists (IFOMPT) recommendations to reduce the risk of adverse effects (Rushton et al., 2017). The training exercises followed the same progression and quantity as the Exercise Group.

All the exercises and manual therapy techniques described have been shown to be effective on chronic neck pain (Fernandez Peñas & Cleland, 2011; Hidalgo García et al., 2016; Malo-Urriés et al., 2017; Carrasco-Uribarren et al., 2020).

### Sample size calculation

The first variable used for sample size calculation in our study was the neck disability index, analyzed according to its disability categories. The sample size calculation was made with G*Power software (Heinrich Heine University Düsseldorf: https://www.jospt.org/action/ecommercehttps://www.psychologie.hhu.de/arbeitsgruppen/allgemeine-psychologie-und-arbeitspsychologie/gpower). Test family: X2 tests. Statistical test: goodness-of-fit test: contingency tables. Type of power analysis: *a priori*: compute required sample size-given alfa, power, and effect size. According to Cohen (1992), the effect size greater than 0.5 calculated from a contingency table with an ordinal and a nominal variable is strong. Previous studies have found an effect size of 1.15 for interventions based on manual therapy plus exercise in patients with chronic neck pain (Celenay, Akbayrak & Kaya, 2016). Thereby, we used an effect size of 1.15 with 12 grades of freedom, an α risk of 0.05 and a β risk of 0.20. The number of subjects needed was 14 per group.

The second variable used for sample size calculation was the flexion-rotation test total ROM. The sample size was calculated using the G*Power software: test family: t tests. Statistical test: means: difference between two independent means (two groups). Type of power analysis: *a priori*: compute required sample size-given alfa, power, and effect size. For calculation, a common standard deviation of 7.18 was used (Dunning et al., 2012) and mean of 14.3 in one group and a mean of six in the other group (Dunning et al., 2012), with an α risk of 0.05, a β risk of 0.20, and test two-side. The number of subjects needed was 13 per group.

To solve possible losses to follow-up, the sample size of each of the groups was increased to 29 subjects.

### Statistical analysis

The statistical analysis was conducted using SPSS 25.0 package (IBM, Armonk, New York, NY, USA) to assess group differences in neck disability index, flexion-rotation test, pressure pain threshold, cervical range of motion, and pain intensity of success at each time interval.

**Figure 3:**
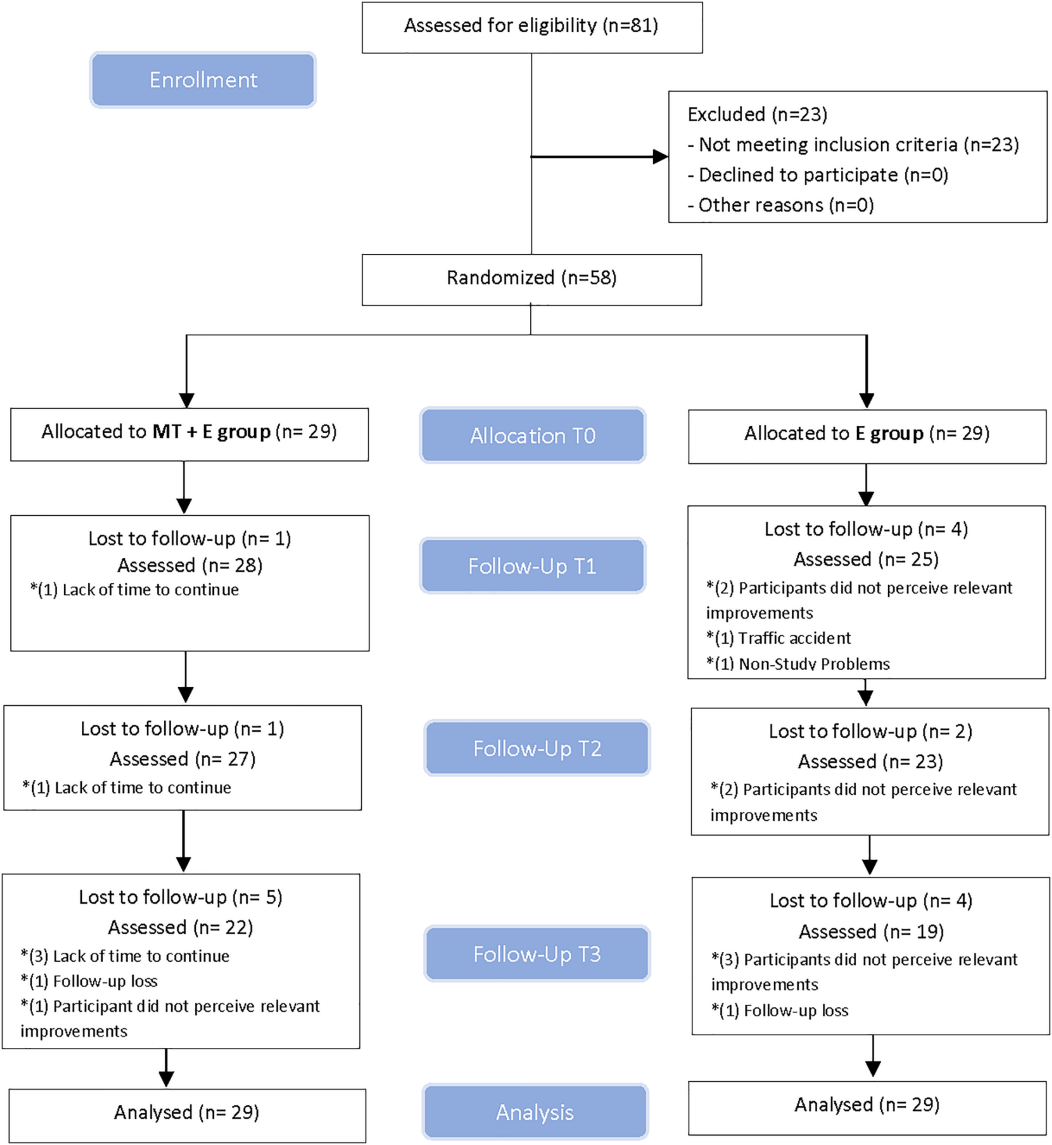
CONSORT. (Consolidated standards of reporting trial) flow diagram.

The linear mixed model was used to compare between-group and within-group changes over the four measurement periods. This model was performed for each dependent variable where the treatment group (MT+Exercise or E) was the between-subjects factor, and time was the within-subjects factor. The Shapiro-Wilk test was used to determine a normal distribution of quantitative data (*p* > 0.05). Outliers were examined. No value was excluded because extreme values did not cause any significant bias. If the assumption of sphericity was violated, the Greenhouse-Geisser correction was utilized for interpretation. When a statistically significant effect was noted, a post-hoc analysis was performed and the Bonferroni correction was used to adjust for multiple comparisons (Reynolds et al., 2020). For the neck disability index variable, a likelihood-ratio chi-square test was used. Effect sizes were calculated using Cohen’s d coefficient (Cohen, 1988) for quantitative variables. An effect size > 0.8 was considered large; around 0.5, intermediate; and <0.2, small (Cohen, 1988). For qualitative variables, Cramer’s V was used to calculate the effect size (Cohen, 1988). An effect size > 0.5 was considered strong; between 0.5–0.3, intermediate; and <0.3, a small effect (Cohen, 1988). All subjects originally enrolled were included in the final analysis as planned. Losses and exclusions after randomization are explained in Fig. 3. The statistical analysis was performed on an intention-to-treat basis (Little’s missing completely at random test and expectation maximization). The level of significance was set at *p* < 0.05.

## Results

Eighty-one volunteers were assessed for eligibility between October 2018 and January 2020. Fifty-eight participants (17 men and 41 women), with a mean age of 49.2 ± 15.9 years, met all eligibility criteria and agreed to participate. Participants were randomly assigned into two groups of 29, they received their assigned treatment and were analyzed for intention-to-treat. Drop-outs and enrollment exclusions before randomization and follow-ups are displayed in a flow diagram, see Fig. 3. The demographic characteristics of the sample are summarized in Table 1. There were no adverse events from the treatments performed in the study at any follow-up.

**Table 1:** Baseline features for both groups.

	Exercise group (*n* = 29)	MT+Exercise group (*n* = 29)
**Clinical features**		
Age, (years)	49.72 ± 17.56	48.76 ± 14.53
Sex (female)	22 (75.9%)	19 (65.5%)
Duration of symptoms, months	124.38 ± 148.17	96.97 ± 96.73
**Neck disability index**		
No disability	0 (0%)	3 (10.3%)
Mild disability	16 (55.2%)	17 (58.6%)
Moderate disability	10 (34.5%)	8 (27.6%)
Severe disability	3 (10.3%)	1 (3.4%)
Complete disability	0 (0%)	0 (0%)
**Flexion-rotation test** (°)		
Right	16.70 ± 9.52	21.26 ± 10.71
Left	19.01 ± 10.33	23.12 ± 8.98
**Cervical ROM** (°)		
Flexion	48.10 ± 10.93	47.48 ± 12.85
Extension	51.48 ± 12.66	53.59 ± 14.36
Lateral flexion (right)	27.97 ± 8.59	32.03 ± 9.93
Lateral flexion (left)	29.38 ± 9.12	30.28 ± 9.83
Rotation (right)	53.97 ± 12.32	55.66 ± 16.07
Rotation (left)	55.28 ± 14.34	58.14 ± 16.37
**Pain intensity NPRS** (0–10)	4.28 ± 2.48	4.10 ± 1.70
**Pressure pain threshold** (kPa)		
Trapezius (right)	192.17 ± 88.42	208.00 ± 98.75
Levator scapulae (right)	180.69 ± 105.62	213.45 ± 132.29
C5-6 (right)	152.86 ± 63.17	177.59 ± 84.66
Trapezius (left)	213.28 ± 97.49	237.97 ± 113.66
Levator scapulae (left)	190.24 ± 122.36	223.62 ± 141.34
C5-6 (left)	153.90 ± 72.56	175.76 ± 76.25

**Note:**

MT+Exercise, Manual Therapy+Exercise; M, Male; ROM, Range of Motion; NPRS, Numerical Pain Rating Scale.

**Figure 4:**
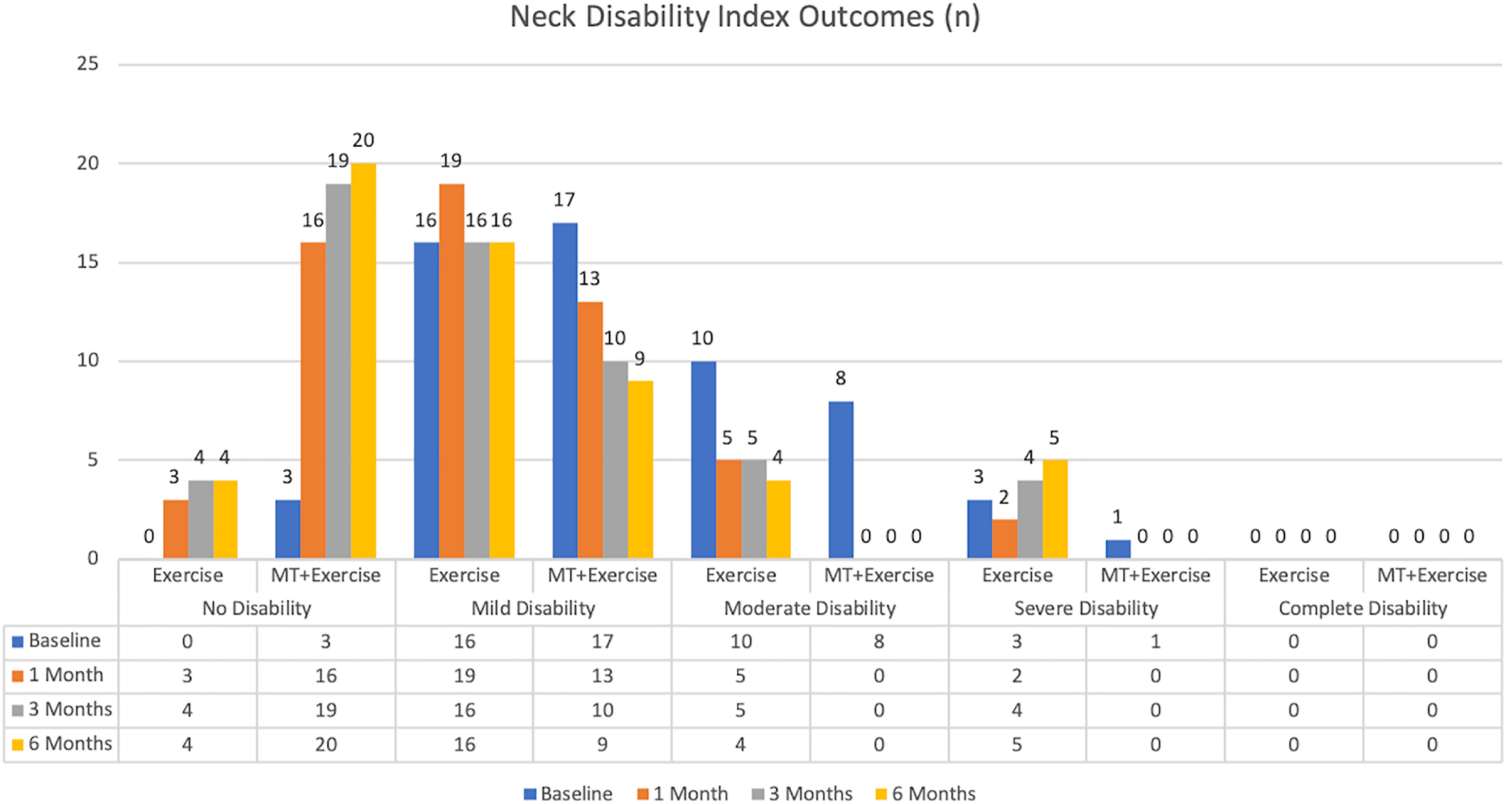
Neck disability index outcomes.

We found statistically significant differences between-groups in the neck disability index favoring the MT+Exercise group in T1, T2, and T3 (*p* = 0.000) (Table 2) (Fig. 4).

**Table 2:** Outcomes neck disability index values between group.

Neck disability index	Group	T0	T1			T2			T3		
		Baseline	1 Month		Difference between groups	3 Months		Difference between groups	6 Months		Difference between groups
		N (%)	N (%)	(%)	p and r value	N (%)	(%)	p and r value	N (%)	(%)	*p* and r value
**No disability**	Exercise	0 (0%)	3 (10.3%)	44.9%	***p* = 0.000** *^C^* **r = 0.54** *^V^*	4 (13.8%)	51.7%	***p* = 0.000** *^C^* **r = 0.59** *^V^*	4 (13.8%)	51.7%	***p* = 0.000** *^C^* **r = 0.61** *^V^*
	MT + Exercise	3 (10.3%)	16 (55.2%)			19 (65.5%)			20 (69%)		
**Mild disability**	Exercise	16 (55.2%)	19 (65.5%)	24.1%		16 (55.2%)	24.2%		16 (55.2%)	20.7%	
	MT + Exercise	17 (58.6%)	13 (44.8%)			10 (34.5%)			9 (31%)		
**Moderate disability**	Exercise	10 (34.5%)	5 (17.2%)	17.2%		5 (17.2%)	17.2%		4 (13.8%)	17.2%	
	MT + Exercise	8 (27.6%)	0 (0%)			0 (0%)			0 (0%)		
**Severe disability**	Exercise	3 (10.3%)	2 (6.9%)	3.5%		4 (13.8%)	10.4%		5 (17.2%)	13.8%	
	MT + Exercise	1 (3.4%)	0 (0%)			0 (0%)			0 (0%)		
**Complete disability**	Exercise	0 (0%)	0 (0%)	0%		0 (0%)	0%		0 (0%)	0%	
	MT + Exercise	0 (0%)	0 (0%)			0 (0%)			0 (0%)		

**Notes:**

C MT + Exercise, Manual Therapy + Exercise; Likelihood-ratio Chi-square test.

V Cramer‘s V; bold *p* values ≤ 0.05 are statistically significant.

Descriptive values of central tendency and dispersion for all quantitative variables, in each group, can be consulted in Table 3.

**Table 3:** Descriptive values of central tendency and dispersion by group.

Measurement	MT+Exercise group Mean ± SD	Exercise group Mean ± SD	
**Right flexion-rotation Test (°)**			
Baseline (T0)	21.26 ± 10.71	16.70 ± 9.52	
End of Intervention (T1)	40.89 ± 8.63	18.56 ± 12.46	
3 Months (T2)	37.76 ± 9.25	14.56 ± 8.27	
6 Months (T3)	37.94 ± 9.64	14.56 ± 8.76	
**Left flexion-rotation test (°)**			
Baseline (T0)	23.12 ± 8.98	19.01 ± 10.33	
End of intervention (T1)	39.49 ± 10.47	20.57 ± 12.05	
3 Months (T2)	38.81 ± 7.41	15.17 ± 7.41	
6 Months (T3)	37.32 ± 9.32	15.53 ± 11.78	
**Flexion ROM (°)**			
Baseline (T0)	47.48 ± 12.85	48.10 ± 10.93	
End of intervention (T1)	47.84 ± 10.25	51.11 ± 12.20	
3 Months (T2)	51.64 ± 8.04	48.89 ± 10.70	
6 Months (T3)	54.43 ± 9.57	46.34 ± 8.88	
**Extension ROM (°)**			
Baseline (T0)	53.59 ± 14.36	51.48 ± 12.66	
End of intervention (T1)	57.24 ± 14.16	52.78 ± 12.58	
3 Months (T2)	60.30 ± 17.17	52.61 ± 9.65	
6 Months (T3)	63.59 ± 10.26	54.85 ± 10.64	
**Right lateral flexion ROM (°)**			
Baseline (T0)	32.03 ± 9.93	27.97 ± 8.59	
End of intervention (T1)	31.75 ± 9.61	28.73 ± 9.48	
3 Months (T2)	33.97 ± 9.99	29.03 ± 9.60	
6 Months (T3)	38.21 ± 8.06	29.58 ± 8.29	
**Left lateral flexion ROM (°)**			
Baseline (T0)	30.28 ± 9.83	29.38 ± 9.12	
End of intervention (T1)	33.12 ± 9.42	28.93 ± 10.06	
3 Months (T2)	35.50 ± 9.01	29.61 ± 7.45	
6 Months (T3)	39.49 ± 8.13	31.43 ± 9.43	
**Right rotation ROM (°)**			
Baseline (T0)	55.66 ± 16.07	53.97 ± 12.32	
End of intervention (T1)	59.58 ± 14.32	55.98 ± 13.49	
3 Months (T2)	66.89 ± 13.65	56.92 ± 13.17	
6 Months (T3)	70.34 ± 10.72	58.72 ± 12.32	
**Left rotation ROM (°)**			
Baseline (T0)	58.14 ± 16.37	55.28 ± 14.34	
End of intervention (T1)	62.40 ± 14.77	59.36 ± 14.90	
3 Months (T2)	65.92 ± 14.73	57.77 ± 12.93	
6 Months (T3)	69.41 ± 12.39	59.92 ± 12.17	
**Pain intensity NPRS (0–10)**			
Baseline (T0)	4.10 ± 1.70	4.28 ± 2.48	
End of intervention (T1)	0.70 ± 1.74	3.46 ± 2.25	
3 Months (T2)	1.28 ± 1.63	3.46 ± 2.56	
6 Months (T3)	1.17 ± 1.52	3.91 ± 2.77	
**Right trapezius PPT (kPa)**			
Baseline (T0)	208.00 ± 98.75	192.17 ± 88.42	
End of intervention (T1)	270.31 ± 130.00	182.05 ± 83.57	
3 Months (T2)	257.88 ± 110.01	171.80 ± 75.87	
6 Months (T3)	319.27 ± 124.45	185.73 ± 76.17	
**Right levator scapulae PPT (kPa)**			
Baseline (T0)	213.45 ± 132.29	180.69 ± 105.62	
End of intervention (T1)	255.46 ± 101.46	156.28 ± 76.52	
3 Months (T2)	269.51 ± 132.46	149.03 ± 60.43	
6 Months (T3)	328.26 ± 152.04	155.75 ± 64.38	
**Right C5-6 PPT (kPa)**			
Baseline (T0)	177.59 ± 84.66	152.86 ± 63.17	
End of intervention (T1)	220.81 ± 99.93	146.36 ± 74.12	
3 Months (T2)	239.85 ± 103.77	142.78 ± 63.04	
6 Months (T3)	288.19 ± 110.63	137.56 ± 61.36	
**Left trapezius PPT (kPa)**			
Baseline (T0)	237.97 ± 113.66	213.28 ± 97.49	
End of intervention (T1)	322.72 ± 187.00	197.38 ± 89.16	
3 Months (T2)	292.83 ± 120.36	188.35 ± 99.80	
6 Months (T3)	348.97 ± 151.29	205.81 ± 87.90	
**Left levator scapulae PPT (kPa)**			
Baseline (T0)	223.62 ± 141.34	190.24 ± 122.36	
End of intervention (T1)	269.73 ± 129.89	179.71 ± 89.83	
3 Months (T2)	278.78 ± 115.02	152.50 ± 66.47	
6 Months (T3)	331.57 ± 108.87	166.65 ± 67.31	
**Left C5-6 PPT (kPa)**			
Baseline (T0)	175.76 ± 76.25	153.90 ± 72.56	
End of intervention (T1)	238.16 ± 108.40	155.36 ± 75.31	
3 Months (T2)	255.56 ± 111.16	142.19 ± 60.34	
6 Months (T3)	286.91 ± 122.63	133.34 ± 50.86	

**Note:**

MT+Exercise, Manual therapy+Exercise; ROM, Range of Motion; PPT, Pressure Pain Threshold; NPRS, Numerical Pain Rating Scale.

### Flexion-rotation test

There were significant main effects in FRT right and left for time: right: F = 22.92 (*p* = 0.000); left: F = 20.11 (*p* = 0.000); and for group: right: F = 87.13 (*p* = 0.000); left: F:63.26 (*p* = 0.000). There was also a significant interaction between group and time on the right: F = 23.90 (*p* = 0.000) and left rotation: F = 28.59 (*p* = 0.000). These effects indicated that Exercise Group did not change over time whereas the MT+Exercise Group increased ROM over time (Table 4), and compared to the Exercise Group in the three moments of the study (Table 5).

**Table 4:** Values of the intragroup effect.

Measurement		MT+Exercise group Mean ± SD	*p* value	Exercise group Mean ± SD		*p* value
**Right flexion-rotation test (°)**						
Change from T0 to T1		19.63 ± 11.19	**0.000**	1.84 ± 12.45		1.000
Change from T0 to T2		16.50 ± 12.17	**0.000**	−2.14 ± 9.62		1.000
Change from T0 to T3		16.68 ± 13.34	**0.000**	−2.14 ± 9.21		1.000
**Left flexion-rotation test (°)**						
Change from T0 to T1		16.37 ± 8.83	**0.000**	1.54 ± 8.41		1.000
Change from T0 to T2		15.70 ± 9.11	**0.000**	−3.84 ± 8.58		0.137
Change from T0 to T3		14.20 ± 7.78	**0.000**	−3.46 ± 8.41		0.147
**Flexion ROM (°)**						
Change from T0 to T1		0.34 ± 15.30	1.000	3.01 ± 12.44		1.000
Change from T0 to T2		4.16 ± 14.31	0.456	0.78 ± 10.08		1.000
Change from T0 to T3		6.95 ± 9.38	**0.003**	−1.76 ± 10.69		1.000
**Extension ROM (°)**						
Change from T0 to T1		3.65 ± 9.55	0.327	1.30 ± 10.46		1.000
Change from T0 to T2		6.71 ± 11.08	**0.046**	1.13 ± 14.77		1.000
Change from T0 to T3		10.00 ± 11.89	**0.000**	3.37 ± 12.52		0.857
**Right lateral flexion ROM (°)**						
Change from T0 to T1		−0.28 ± 6.72	1.000	0.76 ± 6.88		1.000
Change from T0 to T2		1.94 ± 7.18	1.000	1.06 ± 8.93		1.000
Change from T0 to T3		6.18 ± 7.68	**0.000**	1.61 ± 7.77		1.000
**Left lateral flexion ROM (°)**						
Change from T0 to T1		2.84 ± 4.43	0.102	−0.45 ± 5.99		1.000
Change from T0 to T2		5.22 ± 6.70	**0.009**	0.23 ± 9.77		1.000
Change from T0 to T3		9.21 ± 7.35	**0.000**	2.05 ± 10.14		1.000
**Right rotation ROM (°)**						
Change from T0 to T1		3.92 ± 11.07	0.429	2.01 ± 11.93		1.000
Change from T0 to T2		11.24 ± 13.33	**0.000**	2.95 ± 14.01		1.000
Change from T0 to T3		14.68 ± 12.37	**0.000**	4.75 ± 11.96		0.240
**Left rotation ROM (°)**						
Change from T0 to T1		3.94 ± 11.08	0.309	4.08 ± 12.07		0.369
Change from T0 to T2		11.22 ± 13.33	**0.022**	2.49 ± 14.72		1.009
Change from T0 to T3		14.67 ± 12.37	**0.000**	4.64 ± 12.29		0.292
**Pain intensity NPRS (0-10)**						
Change from T0 to T1		−3.40 ± 2.41	**0.000**	−0.82 ± 2.72		0.548
Change from T0 to T2		−2.82 ± 2.05	**0.000**	−0.82 ± 3.17		0.631
Change from T0 to T3		−2.93 ± 1.88	**0.000**	−0.37 ± 2.84		1.000
**Right trapezius PPT (kPa)**						
Change from T0 to T1		62.31 ± 90.25	**0.000**	−10.12 ± 57.84		1.000
Change from T0 to T2		49.88 ± 114.70	**0.048**	−20.37 ± 76.72		1.000
Change from T0 to T3		111.27 ± 100.57	**0.000**	−6.44 ± 96.60		1.000
**Right levator scapulae PPT (kPa)**						
Change from T0 to T1		42.01 ± 85.73	**0.042**	−24.41 ± 75.44		0.655
Change from T0 to T2		56.06 ± 132.80	**0.107**	−31.66 ± 113.77		1.000
Change from T0 to T3		114.81 ± 125.67	**0.000**	−24.96 ± 125.08		1.000
**Right C5-6 PPT (kPa)**						
Change from T0 to T1		43.22 ± 75.51	**0.004**	−6.50 ± 53.72		1.000
Change from T0 to T2		62.26 ± 83.86	**0.000**	−10.08 ± 69.54		1.000
Change from T0 to T3		110.60 ± 90.09	**0.000**	−15.30 ± 76.56		1.000
**Left trapezius PPT (kPa)**						
Change from T0 to T1		84.75 ± 121.11	**0.000**	−15.90 ± 79.04		1.000
Change from T0 to T2		54.86 ± 111.10	**0.030**	−24.93 ± 90.37		1.000
Change from T0 to T3		111.00 ± 127.70	**0.000**	−7.47 ± 100.05		1.000
**Left levator scapulae PPT (kPa)**						
Change from T0 to T1		46.11 ± 112.61	**0.081**	−10.53 ± 79.22		1.000
Change from T0 to T2		55.14 ± 110.43	**0.090**	−37.74 ± 125.59		0.547
Change from T0 to T3		107.95 ± 117.46	**0.000**	−23.59 ± 135.18		1.000
**Left C5-6 PPT (kPa)**						
Change from T0 to T1		62.40 ± 90.15	**0.000**	1.46 ± 60.32		1.000
Change from T0 to T2		79.80 ± 94.90	**0.000**	−11.69 ± 72.33		1.000
Change from T0 to T3		111.15 ± 110.68	**0.000**	−20.56 ± 77.17		1.000

**Notes:**

MT+Exercise, Manual therapy+Exercise; ROM, Range of Motion; PPT, Pressure Pain Threshold; NPRS, Numerical Pain Rating Scale.

Bold *p* values ≤ 0.05 are statistically significant.

**Table 5:** Values of the intergroup effect.

Measurement	Between-group differences^†^	*p* value	Effect size
**Right flexion-rotation test (°)**			
Change from T0 to T1	17.79 (6.49–14.99)	**0.000**	0.529
Change from T0 to T2	18.64 (3.24–11.12)	**0.000**	0.644
Change from T0 to T3	18.82 (3.15–11.38)	**0.000**	0.625
**Left Flexion-Rotation Test (°)**			
Change from T0 to T1	14.83 (5.68–12.06)	**0.000**	0.421
Change from T0 to T2	19.54 (2.75–9.10)	**0.000**	0.725
Change from T0 to T3	17.66 (2.45–8.27)	**0.000**	0.521
**Flexion ROM (°)**			
Change from T0 to T1	−2.07 (−3.33 to 6.69)	0.274	0.021
Change from T0 to T2	3.38 (−1.97 to 6.91)	0.274	0.021
Change from T0 to T3	8.71 (−1.08 to 6.26)	**0.002**	0.166
**Extension ROM (°)**			
Change from T0 to T1	2.35 (−1.12 to 6.07)	0.210	0.028
Change from T0 to T2	5.58 (−0.77 to 8.61)	0.**040**	0.073
Change from T0 to T3	6.63 (2.30 to 11.07)	**0.002**	0.153
**Right Lateral Flexion ROM (°)**			
Change from T0 to T1	−1.04 (−2.20 to 2.68)	0.234	0.025
Change from T0 to T2	0.88 (−1.41 to 4.41)	**0.060**	0.062
Change from T0 to T3	4.57 (1.12 to 6.67)	**0.000**	0.224
**Left Lateral Flexion ROM (°)**			
Change from T0 to T1	3.29 (−1.04 to 3.43)	0.108	0.046
Change from T0 to T2	4.99 (−0.29 to 5.73)	**0.009**	0.116
Change from T0 to T3	7.16 (2.45–8.81)	**0.001**	0.178
**Right Rotation ROM (°)**			
Change from T0 to T1	1.91 (−1.17–7.11)	0.328	0.017
Change from T0 to T2	8.29 (2.19–12.01)	**0.006**	0.125
Change from T0 to T3	9.93 (5.35–14.09)	**0.000**	0.208
**Left Rotation ROM (°)**			
Change from T0 to T1	−0.14 (0.32–8.32)	0.439	0.011
Change from T0 to T2	8.73 (0.17–10.10)	**0.029**	0.082
Change from T0 to T3	10.03 (3.50–12.42)	**0.005**	0.134
**Pain Intensity NPRS (0-10)**			
Change from T0 to T1	−2.58 (−3.03 to −1.19)	**0.000**	0.327
Change from T0 to T2	−2.00 (−2.78 to −0.86)	**0.000**	0.211
Change from T0 to T3	−2.56 (−2.52 to −0.78)	**0.000**	0.280
**Right Trapezius PPT (kPa)**			
Change from T0 to T1	72.43 (−1.13 to 53.32)	**0.003**	0.144
Change from T0 to T2	70.25 (−20.29 to 49.80)	**0.001**	0.177
Change from T0 to T3	117.71 (17.00–87.83)	**0.000**	0.303
**Right Levator Scapulae PPT (kPa)**			
Change from T0 to T1	66.42 (−20.20 to 37.80)	**0.000**	0.240
Change from T0 to T2	87.72 (−32.21 to 56.61)	**0.000**	0.262
Change from T0 to T3	139.77 (−0.09 to 89.96)	**0.000**	0.361
**Right C5-6 PPT (kPa)**			
Change from T0 to T1	49.72 (−4.97 to 41.69)	**0.002**	0.156
Change from T0 to T2	72.34 (−1.58 to 53.76)	**0.000**	0.249
Change from T0 to T3	125.90 (17.63–77.68)	**0.000**	0.423
**Left Trapezius PPT (kPa)**			
Change from T0 to T1	100.65 (−2.30 to 71.16)	**0.002**	0.159
Change from T0 to T2	79.79 (−21.40 to 51.34)	**0.001**	0.188
Change from T0 to T3	118.47 (10.57–92.97)	**0.000**	0.257
**Left Levator Scapulae PPT (kPa)**			
Change from T0 to T1	56.64 (−17.18 to 52.76)	**0.003**	0.144
Change from T0 to T2	92.88 (−33.76 to 51.18)	**0.000**	0.319
Change from T0 to T3	131.54 (−3.30 to 87.66)	**0.000**	0.462
**Left C5-6 PPT (kPa)**			
Change from T0 to T1	60.94 (4.39–59.48)	**0.001**	0.169
Change from T0 to T2	91.49 (3.74–64.35)	**0.000**	0.294
Change from T0 to T3	131.71 (11.03–79.56)	**0.000**	0.409

**Notes:**

ROM, Range of Motion; PPT, Pressure Pain Threshold; NPRS, Numerical Pain Rating Scale.

† values are mean adjusted change scores (95% confidence interval); Between-Group Differences are calculated as Change in MT+E group minus Change in Exercise group.

Bold *p* values ≤ 0.05 are statistically significant.

### Pressure pain threshold

We found significant main effects on the right trapezius PPT for time: F = 7.34 (*p* = 0.000) and for group: F = 13.24 (*p* = 0.001). There was also a significant interaction between group and time: F = 8.84 (*p* = 0.000). These effects indicated that the Exercise Group did not change over time however MT+Exercise Group increased PPT over time (Table 4), and compared to the Exercise Group in the three moments of the study (Table 5).

We found significant main effects on the right levator scapulae PPT for time: F = 4.36 (*p* = 0.014) and for group: F = 20.96 (*p* = 0.000). There was also a significant interaction between group and time: F = 9.46 (*p* = 0.000). These effects indicated that the Exercise Group did not change over time, whereas the MT+Exercise Group increased PPT over time (Table 4), and compared to Exercise Group in the three moments of the study (Table 5).

We found significant main effects on the right C5-6 PPT for time: F = 9.13 (*p* = 0.000) and for group: F = 20.50 (*p* = 0.000). There was also a significant interaction between group and time: F = 16.00 (*p* = 0.000). These effects indicated that the Exercise Group did not change over time whereas the MT+Exercise group increased PPT over time (Table 4), and compared to the Exercise Group in the three moments of the study (Table 5).

We found significant main effects on the left trapezius PPT for time: F = 4.92 (*p* = 0.004) and for group: F = 13.61 (*p* = 0.001). There was also a significant interaction between group and time: F = 6.58 (*p* = 0.001). These effects indicated that the Exercise Group did not change over time, whereas the MT+Exercise Group increased PPT over time (Table 4), and compared to the Exercise Group in the three moments of the study (Table 5).

We found significant main effects on the left levator scapulae PPT for time: F = 3.74 (*p* = 0.012) and for group: F = 19.74 (*p* = 0.000). There was also a significant interaction between group and time: F = 8.85 (*p* = 0.000). These effects indicated that the Exercise Group did not change over time, whereas the MT+Exercise Group increased PPT over time (Table 4), and compared to the Exercise Group in the three moments of the study (Table 5).

We found significant main effects on the left C5-6 PPT for time: F = 6.86 (*p* = 0.000) and for group: F = 23.32 (*p* = 0.000). There was also a significant interaction between group and time: F = 13.97 (*p* = 0.000). These effects indicated that the Exercise Group did not change over time, whereas the MT+Exercise Group increased PPT over time (Table 4), and compared to the Exercise Group in the three moments of the study (Table 5).

### Cervical range of motion

We found a significant interaction on flexion between group and time: F = 5.54 (*p* = 0.003). This interaction indicated that the Exercise group did not change over time, whereas the MT+Exercise Group increased the ROM at 6 months (Table 4), and compared to Exercise group at 6 months (Table 5).

There was a significant main effect on extension for time: F = 6.99 (*p* = 0.000) and for group: F = 4.06 (*p* = 0.049). This effect indicated that the Exercise Group did not change over time, whereas the MT+Exercise Group increased the ROM at 3 and 6 months (Table 4), and compared to the Exercise Group at 3 and 6 months.

We found significant main effects on right lateral flexion ROM for time: F = 7.80 (*p* = 0.000) and for group: F = 5.75 (*p* = 0.020). There was also a significant interaction between group and time: F = 3.66 (*p* = 0.019). These effects indicated that the Exercise group did not change over time, whereas the MT+Exercise Group increased the ROM at 6 months (Table 4), and compared to the Exercise Group at 3 and 6 months (Table 5).

We found significant main effects on left lateral flexion ROM for time: F = 12.66 (*p* = 0.000) and for group: F = 5.27 (*p* = 0.026). There was also a significant interaction between group and time: F = 4.88 (*p* = 0.006). These effects indicated that the Exercise Group did not change over time, whereas the MT+Exercise Group increased the ROM at 3 and 6 months (Table 4), and compared to the Exercise Group at 3 and 6 months (Table 5).

We found significant main effects on right rotation ROM for time: F = 16.37 (*p* = 0.000) and for group: F = 5.10 (*p* = 0.028). There was also a significant interaction between group and time: F = 5.11 (*p* = 0.006). These effects indicated that the Exercise Group did not change over time, whereas the MT+Exercise Group increased the ROM at 3 and 6 months (Table 4), and compared to the Exercise Group at 3 and 6 months (Table 5).

There was significant main effect on left rotation ROM for time: F = 10.41 (*p* = 0.000), with almost a significant interaction effect for time and group: F = 2.83 (*p* = 0.051). This effect indicated that Exercise group did not change over time, whereas the MT+Exercise Group increased the ROM at 3 and 6 months (Table 4), and compared to the Exercise Group at 3 and 6 months (Table 5).

### Pain intensity (NPRS)

**Figure 5:**
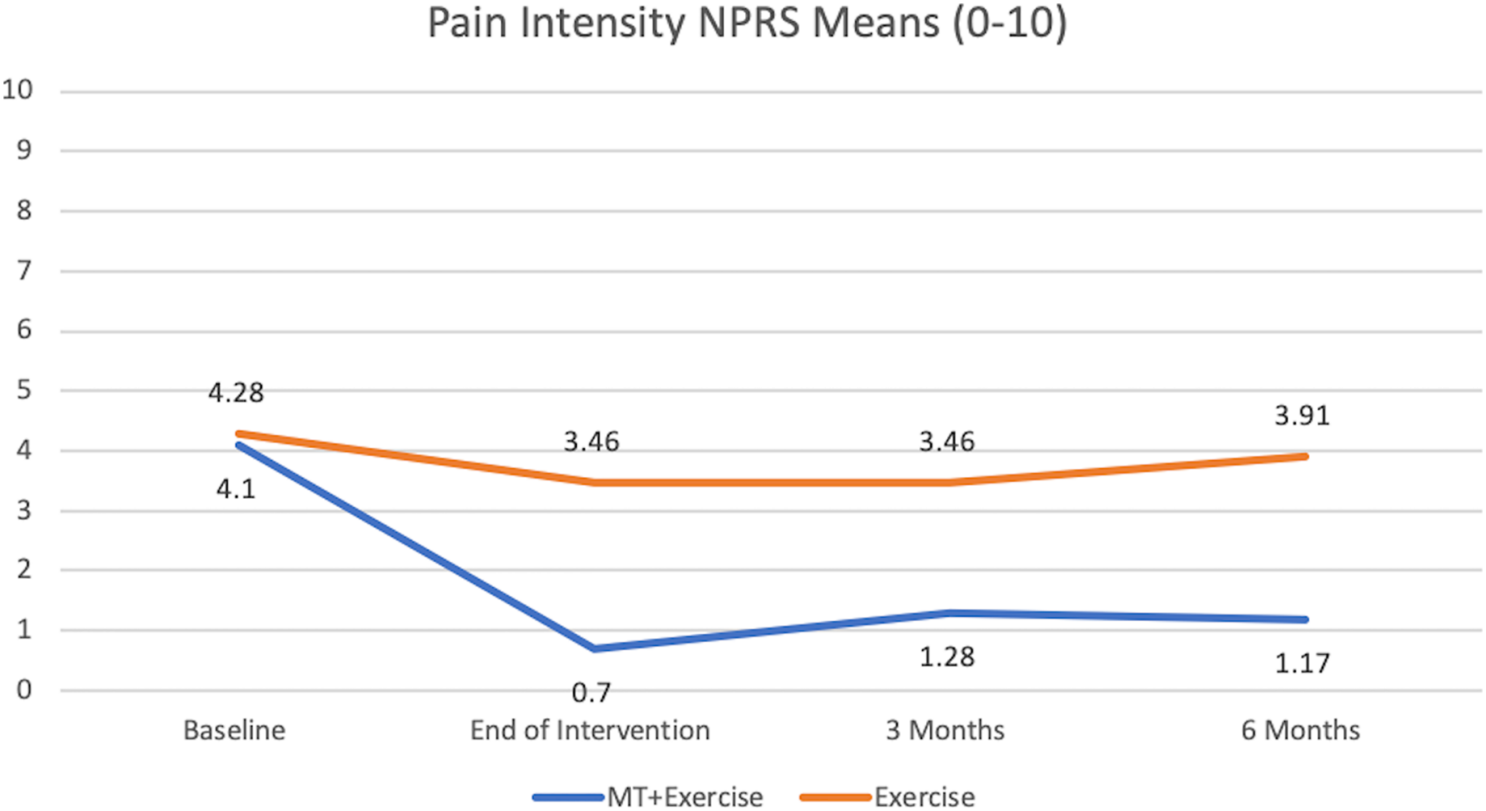
Pain intensity NPRS means (0–10).

We found significant main effects on the NPRS for time: F = 17.58 (*p* = 0.000) and for group: F = 24.15 (*p* = 0.000) (Fig. 5). There was also a significant interaction between group and time: F = 7.27 (*p* = 0.000). These effects indicated that the Exercise Group did not change over time, whereas the MT+Exercise Group decreased pain intensity over time (Table 4), and compared to Exercise Group at the end of intervention, at 3 and 6 months (Table 5).

Three Exercise group participants reported mild and transient aggravation of neck pain in the 6-month follow-up.

## Discussion

The present study has found statistically significant differences in the results obtained when complementing neck exercises with manual therapy in the treatment of chronic neck pain and upper cervical rotation restriction. Although these results are in line with some previous research (Izquierdo Pérez et al., 2014; Celenay, Akbayrak & Kaya, 2016; Cho, Lee & Lee, 2017), the improvements found in the MT+Exercise Group under analysis were greater than those found in earlier studies.

In the neck disability index, different studies support the use of cervical exercise leading to positive results in patients’ disability (Miller et al., 2010; Falla et al., 2013; Gallego Izquierdo et al., 2016; Treleaven et al., 2016). However, the improvement in the MT+Exercise Group was higher than the Exercise Group in our study and greater than those found in earlier studies (Izquierdo Pérez et al., 2014; Celenay, Akbayrak & Kaya, 2016; Cho, Lee & Lee, 2017). At baseline, 31% of the patients in the MT+Exercise Group had moderate, severe or complete disability and after intervention, none of them did. At 3 and 6 months, 31% of the patients in Exercise Group still had moderate or severe disability. A classification in NDI, under moderate disability, has been shown to predict avoidance of chronic disability after whiplash in 55.7% of the patients (Nederhand et al., 2004). A recent study, which compared the efficacity of manual therapy *vs* exercise in chronic neck pain, has shown an effect side at three months of 0.18 in the NDI, which is less than the 0.59 obtained in the current study (Bernal-Utrera et al., 2020).

The MT+Exercise Group was more effective than the Exercise Group, increasing right and left flexion-rotation tests and active cervical range of motion. The manual therapy approach, aimed at restoring the mobility of the upper cervical joints, has shown to improve values in the flexion-rotation test by treating C0-1 (Hidalgo García et al., 2016; Malo-Urriés et al., 2017; González-Rueda et al., 2020), C2-3 (Carrasco-Uribarren et al., 2020), and C1-2 (Hidalgo García et al., 2016), when necessary. This test measures the upper cervical rotation, mainly in C1-2 segment (Takasaki et al., 2011). Consequently, this study followed the international safety recommendations promoting indirect manual therapy treatment of the segment with more dysfunction (C1-2), and avoiding end-range procedures (Rushton et al., 2014). Patients with an upper cervical rotation restriction may have had a restricted cervical range of motion due to the interaction of the upper cervical spine within the cervical spine (Hidalgo-García et al., 2015). Articular or muscular upper cervical spine restriction would affect the degree of freedom of movement of the lower cervical spine. Lateral flexion is associated with an ipsilateral rotation in the inferior cervical spine, and with a contralateral rotation in the superior cervical spine, therefore, a restricted superior cervical rotation may be related to a lack of movement at contralateral lateral flexion in the lower cervical spine (Hidalgo-García et al., 2015). This could explain why restoring upper cervical rotation in the MT+Exercise Group produced better results on the cervical range of motion at 3 and 6 months. Our study suggests that adding manual therapy to a cervical exercise protocol may yield better results for maintaining an upper cervical rotation over time, and cervical range of motion at a medium and medium-long term follow-up than an exercise program. If we compare the results obtained with other studies, Lluch et al. (2014) divided patients with chronic neck pain into two groups. One group performed cervical training and the other received a C0-C1 dorsal gliding technique (similar to one of the techniques used in this study) and cervical training for 3 min. This author did not observe significant changes in any variable of the lower cervical spine range of motion (*p* = 0.35) in either group. Another study (Izquierdo Pérez et al., 2014) used manual therapy in chronic neck pain obtaining improvements in cervical range on motion in all planes of movement: flexion = 13.2°; extension = 12°; lateral flexion = 7.6°; rotation = 9.5°. These values are much lower than the obtained in our study except for flexion and extension.

Besides, the MT+Exercise Group achieved the MDC in the rotation cervical range of motion (more than 5°–10°) (Fletcher & Bandy, 2008; Audette et al., 2010) and in the flexion-rotation test (more than 4.7°–7°) (Hall et al., 2010) of the CROM device. Another possible explanation for the gain of motion of the lower cervical spine, since this region has not been directly addressed, could be through neurophysiological mechanisms. Various studies have indicated that mobilization or manipulation techniques can reduce muscle spasm by activation of primary efferent fibers of the neuromuscular spindles and Golgi organs (Pickar & Wheeler, 2001; Sung, Kang & Pickar, 2005; Pickar & Kang, 2006; Pickar et al., 2007). In the same way, improved movement in the upper cervical region, and the performance of a painless training could improve the capacity of contraction of the cervical musculature. Several authors have suggested that a manipulation technique may alter the afferent discharge, and stimulate mechanoreceptors and proprioceptors by changing the levels of alpha motor neuron excitability, and hence the muscle activity (Metcalfe, Reese & Sydenham, 2006; Pickar & Kang, 2006; Dunning & Rushton, 2009; Fritz et al., 2011).

However, unlike previous studies (Rushton et al., 2014) we did not find an improvement in cervical mobility in our Exercise Group. Our specific inclusion criteria may explain this difference, as patients with upper cervical rotation restriction could experience greater difficulty in improving their mobility while performing the cervical exercise (Hidalgo-García et al., 2015).

This study found significant differences in pain intensity and pressure pain thresholds favoring the MT+Exercise Group with a large effect size. The MT+Exercise Group achieved the MDC (more than two points) (Cleland, Childs & Whitman, 2008) in pain intensity in all post-intervention follow-ups in this study. Our results regarding pain alleviation are in line with those in other studies that found improvements in cervical pain in MT+Exercise groups (Lluch et al., 2014; Farooq et al., 2018), although studies with an isolated manual therapy approach (Izquierdo Pérez et al., 2014) and isolated exercise (Kim, Choi & Lee, 2016) have also shown pain reduction. In the pressure pain threshold, previous studies found no difference applying manual therapy alone (River, Levital & Belgrade, 2012; Malo-Urriés et al., 2017). Our MT+Exercise intervention was more effective than the Exercise, showing similar results to Celenay, Akbayrak & Kaya (2016), although other researchers did not find any difference between groups (Gallego Izquierdo et al., 2016; Cho, Lee & Lee, 2017). Our results support other studies that suggest that manual therapy provokes hypoalgesia locally and in distant regions from the area of the target treatment (Wright, 1995; Vicenzino, Collins & Wright, 1996; Grant, 2002; Pickar, 2002; Haas et al., 2003), through segmental inhibitory pathways, spinal cord pathways, or descending inhibitory pathways of the brain stem (Vicenzino et al., 2001; Skyba et al., 2003). Additionally, the MT+Exercise Group achieved the MDC over all the cervical spine points (more than 47.2 kilopascales) (Walton et al., 2011).

### Limitations

The results of this study are limited due to a sample design that followed several very specific inclusion and exclusion criteria, which may limit the generalization of the results. A reliability study of the measurements has not been carried out for this study, although the methods carried out have been validated in previous studies. They were performed by the same researcher specialized in physical therapy, with training in the evaluation, and more than 5 years’ clinical experience, The manual therapy approach was adapted to the clinical findings of the upper cervical spine. This clinical approach does not allow one to pinpoint which specific intervention was more effective. Moreover, subjects were periodically asked and supervised regarding the performance of the home-exercises, however, the methodology presented limitations in controlling their actual performance of the home-exercises. Other limitations include the sole practitioner who provided the interventions; therefore, the results may not be generalized to other therapists. The loss of subjects to follow-up resulted in a small sample size that also is a threat to the external validity. According to the reasons expressed by the subjects, the losses to follow-up in the study were due to lack of time or the perception that the improvements obtained were not sufficient to motivate the subject to continue in the study. Future studies should try to solve these difficulties, perhaps by proposing less demanding exercise protocols for the follow-up period.

The clinical implication of this study suggests that patients with upper cervical rotation restriction do not respond in the same way as other patients. In order to obtain optimal results, it is interesting that mobility must be restored using manual therapy before applying the exercises.

## Conclusions

Four 20-min sessions of manual therapy and exercise, along with a home-exercise program, was found to be more effective than an exercise protocol and a home-exercise program in improving the neck disability index, flexion-rotation test, pain intensity, and pressure pain threshold, in the short, medium, and medium-long term in patients with chronic neck pain and upper rotation restriction. Cervical range of motion improved with the addition of manual therapy in the medium and medium-long term. The high dropout rate may have compromised the external validity of the study.

## Supplemental Information

10.7717/peerj.12546/supp-1 Supplemental Information 1 Raw data.

10.7717/peerj.12546/supp-2 Supplemental Information 2 Consort Checklist.

10.7717/peerj.12546/supp-3 Supplemental Information 3 Codebook.

## Additional Information and Declarations

### Data Availability

The following information was supplied regarding data availability: The raw measurements are available in the Supplementary File.

### Clinical Trial Registration

The following information was supplied regarding Clinical Trial registration: NCT03670719.
