# Supplementary material for: Comparison of an exercise program with and without manual therapy for patients with chronic neck pain and upper cervical rotation restriction. Randomized controlled trial
Source: PeerJ. 2021 Nov 24;9:e12546. doi: 10.7717/peerj.12546 (PMC8627131; doi:10.7717/peerj.12546)
Supplement: Supplemental Information 3 [file peerj-09-12546-s003.docx]

**CODEBOOK**

| **Variable** | **1** | **2** | **3** | **4** | **5** |
| --- | --- | --- | --- | --- | --- |
| **Treatment Group** | Manual therapy + Exercise Group | Exercise Group | x | x | x |
| **Sex** | Men | Women |  |  |  |
| **NDI_Baseline** | No disability | Mild disability | Moderate disability | Severe disability | Complete disability |
| **NDI_1month** | No disability | Mild disability | Moderate disability | Severe disability | Complete disability |
| **NDI_3months** | No disability | Mild disability | Moderate disability | Severe disability | Complete disability |
| **NDI_6months** | No disability | Mild disability | Moderate disability | Severe disability | Complete disability |
